# Identification of *Ascaris lumbricoides* Eggs within the Pancreas Using Endoscopic Ultrasound

**DOI:** 10.4269/ajtmh.22-0145

**Published:** 2022-09-06

**Authors:** Morgan Freeman, Mandip Kc, Stuart K. Amateau

**Affiliations:** ^1^ Department of Medicine, University of Minnesota, Minneapolis, Minnesota;; ^2^ Division of Gastroenterology, Hepatology and Nutrition, Department of Medicine, Minneapolis, Minnesota

**Figure 1.**
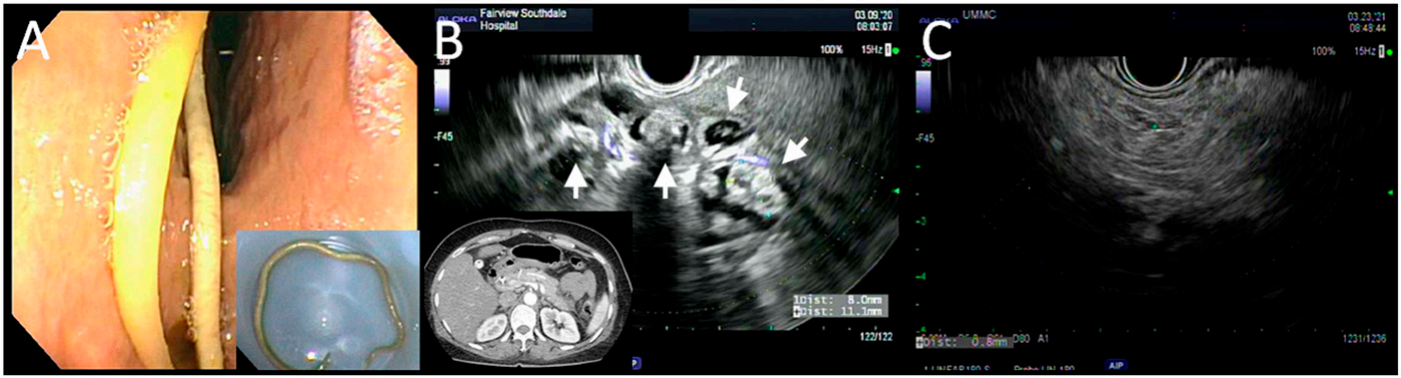
(**A**) Endoscopic visualization of the *Ascaris* worm in the antrum of the stomach. (**B**) Endosonographic images of cystic structures (arrows) adjacent to the tail of the pancreas. (**C**) Repeat endoscopic ultrasound showing resolution of findings after anthelmintic treatment. This figure appears in color at ajtmh.org.

A 50-year-old woman from Micronesia presented with 7 days of progressive nausea, vomiting, and abdominal pain. She had no prior history of hepatobiliary or pancreatic diseases, and she denied alcohol use. Vital signs were within normal limits, and the physical examination was notable for epigastric tenderness. Laboratory investigation revealed a white blood cell count of 19.3 × 10^3^ cells/μL, lipase of 6,499 U/L, aspartate aminotransferase of 308 U/L, alanine aminotransferase of 314 U/L, alkaline phosphatase of 210 U/L, and total bilirubin of 3.8 mg/dL. Abdominal computed tomography showed diffuse, edematous enlargement of the pancreas, along with edema and stranding of surrounding fat, suggesting severe, diffuse acute interstitial pancreatitis. Pronounced intra- and extrahepatic biliary ductal dilation was also noted. During endoscopic ultrasound, white-light evaluation demonstrated an 8-inch roundworm in the antrum of the stomach. It was retrieved in toto and later identified as *Ascaris lumbricoides* (Figure 1A). Endosonographic images showed expected findings of pancreatitis, with the pancreatic parenchyma diffusely and mildly heterogeneous without lobularity; however, there was also a complex region adjacent to the tail of the pancreas with cystic structures (Figure 1B, arrows). These structures were not consistent with necrosis or fluid collections from pancreatitis, and were most likely to be a collection of *Ascaris* eggs, as the initial computed tomographic scan did not show pancreatic stones or other solid structures that would otherwise explain the findings. The patient was initiated on treatment with oral albendazole. Repeat endoscopic ultrasound showed disappearance of the cystic structures (Figure 1C), suggesting that treatment with albendazole led to resolution of the infection.

Ascariasis is one of the most common parasitic infections worldwide. There are an estimated 1.2 million cases of ascariasis in the Oceania region (population, 35 million), which includes Micronesia.^1^^–^^3^ Our patient had last visited Micronesia 4 months earlier. The roundworm is known to lay eggs in the small intestine most commonly.^1^^,^^2^ Migration of worms into the biliary and pancreatic ducts is rare, but well described to cause biliary colic, cholangitis, and acute pancreatitis.^4^^,^^5^ We hypothesize that the roundworm migrated into the pancreatic duct and laid eggs. The eggs are microscopic in size; however, one adult worm lays an estimated 200,000 eggs per day.^6^ A cluster of eggs would likely be seen with endoscopic ultrasound. To our knowledge, this is the first report of *A. lumbricoides* egg deposition identified by endoscopic ultrasound within the pancreas.
